# The Role of Visual Information in Numerosity Estimation

**DOI:** 10.1371/journal.pone.0037426

**Published:** 2012-05-17

**Authors:** Titia Gebuis, Bert Reynvoet

**Affiliations:** 1 Laboratory of Experimental Psychology, University of Leuven, Leuven, Belgium; 2 Subfaculty of psychology and educational sciences, Kulak, University of Leuven, Leuven, Belgium; Cuban Neuroscience Center, Cuba

## Abstract

Mainstream theory suggests that the approximate number system supports our non-symbolic number abilities (e.g. estimating or comparing different sets of items). It is argued that this system can extract number independently of the visual cues present in the stimulus (diameter, aggregate surface, etc.). However, in a recent report we argue that this might not be the case. We showed that participants combined information from different visual cues to derive their answers. While numerosity comparison requires a rough comparison of two sets of items (smaller versus larger), numerosity estimation requires a more precise mechanism. It could therefore be that numerosity estimation, in contrast to numerosity comparison, might rely on the approximate number system. To test this hypothesis, we conducted a numerosity estimation experiment. We controlled for the visual cues according to current standards: each single visual property was not informative about numerosity. Nevertheless, the results reveal that participants were influenced by the visual properties of the dot arrays. They gave a larger estimate when the dot arrays consisted of dots with, on average, a smaller diameter, aggregate surface or density but a larger convex hull. The reliance on visual cues to estimate numerosity suggests that the existence of an approximate number system that can extract numerosity independently of the visual cues is unlikely. Instead, we propose that humans estimate numerosity by weighing the different visual cues present in the stimuli.

## Introduction

The predominant theory in numerical cognition states that we are equipped with an approximate number system that supports our non-symbolic number processes such as estimating or comparing different sets of items. This approximate number system would enable us to extract numerosity from a visual scene (e.g. an array of dots) independently of the visual cues present in that scene (aggregate surface, diameter of the dots, etc.) [1], [2], [3], [4], [5]. This notion is supported by studies that show that humans can perform numerosity comparisons while controlling for visual cues [2], [6], [7]. Visual cues for sets of dots are manipulated and made uninformative of numerosity across trials. Controlling for information other than numerosity is logical if you want to study ‘pure numerosity processes’. But what if numerosity judgments are based on the combination of different visual cues? Two sets of items can differ in numerosity only if their visual characteristics differ, otherwise both would represent the same numerosity. In other words, the only aspect that allows us to dissociate different numbers of objects, are the visual cues present in the stimuli.

Numerosity and visual cues are also highly correlated in real life. For example, when more apples are added to a pile of apples, the size of the pile increases; or when more people enter a room, the density increases. We argue it would therefore be inefficient not to rely on this visual information for numerosity comparison or estimation. In a previous study, we indeed showed that subjects combine information from different visual cues when they have to decide which dot-array contains more dots [8], [9], [10], [11], [12], [13], [14], [15]. This result suggests that current methods control for the visual cues insufficiently as they only control a single visual variable at a time [16], [17]. It also suggests that the existence of a system that can extract numerosity independently of the visual cues is unlikely. For numerosity comparison you only have to make smaller-larger judgments, which can easily be made on the basis of the visual cues present in the stimulus. This visual comparison process is more difficult when visual cues are controlled for. However, despite a decrease in performance [18], [19] participants are still able to perform the task since not all visual cues are controlled for at the same time.

While numerosity comparison processes only require a rough estimate of numerosity, more precise numerosity processes are necessary to estimate the number of items in a set. Izard & Dehaene [3] showed that participants perform poorly when asked to estimate numerosity. In their study, participants highly underestimated the number of dots presented on a screen. The visual cues of dot-arrays were controlled for and post-hoc analyses showed that the participants did not base their judgments on the visual cues present in the stimuli. However, as the authors themselves also suggested, their method for controlling the visual cues of the dot arrays is valid only when a *single* visual variable is used, not when participants combine *multiple* visual cues. The authors did not test whether reliance on multiple visual cues could explain their data. We can therefore not yet conclude that numerosity estimates are conducted independently of the visual cues present in the dot arrays. Our recent results from numerosity comparison suggest that numerosity judgments cannot be performed independently of the visual properties of the stimuli [8]. It is the aim of the present study to test whether this is also true for numerosity estimation.

**Figure 1:**
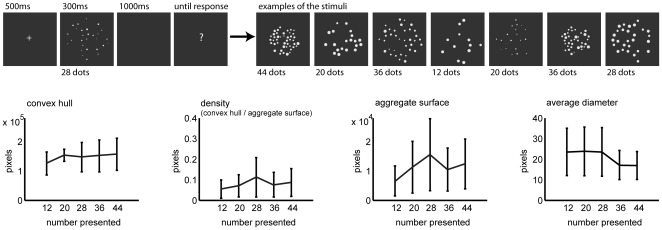
Examples of the stimuli and their visual properties. The upper row represents the task and examples of the stimuli used. The bottom row represents the average visual properties of the different visual cues comprising the dot arrays. Each single visual cue did not systematically increase or decrease with numerosity.

To test the influence of visual cues on numerosity estimation, we presented participants with arrays of dots (12, 18, 24, 36 or 48 dots). The participants were asked to estimate the number of dots presented on a screen. Importantly, we controlled the visual cues of the dot arrays: the size of each visual cue did not systematically increase or decrease with increasing numerosity. For example, the aggregate surface of 28 dots was on average smaller than the aggregate surface of 20 dots but on average larger than the aggregate surface of 36 dots (see Figure 1). Thus, the visual cues were not informative about numerosity across trials. The fact that we controlled for the visual properties is an important difference between this and previous studies investigating the effect of visual cues on numerosity estimation [20], [21]. In this study, there was no incentive for the participant to take the different visual cues into account. In contrast, in previous studies, visual cues correlated with numerosity across trials. If we are equipped with an approximate number system, the participants' numerosity estimates should not be affected by visual cues present in the stimuli. However, if humans cannot extract numerosity independently of visual cues but rely on the sensory input to judge numerosity instead, participants' estimates will show biases induced by the size of the different visual cues.

## Methods

### Participants

Twenty-nine participants (aged between 19 and 30 years) participated in the experiment. No participant was excluded from the analyses. They all had normal or corrected-to-normal vision.

### Ethics statement

Written informed consent was obtained according to the Declaration of Helsinki and as approved by the Ethical Committee of the University of Leuven.

## Materials

The stimuli were arrays of grey dots presented on a dark background (dot size ranged between 0.11 and 0.79 degrees visual angle). The stimuli were generated using a modified version of the program developed by Gebuis & Reynvoet [17]. Each trial consisted of a dot array representing 12, 20, 28, 36 or 44 dots. We used 5 different numerosity values to create a large enough diversity in the stimuli while still being able to control for the visual cues.

We controlled the visual cues to account for the strong correlation between numerosity and its visual properties (when numerosity increases also its visual properties increase). To this end, we manipulated the different visual properties of the numerosity stimuli in such a manner that each single visual property did not consistently increase or decrease with increasing numerosity (see Figure 1). As a consequence of this manipulation, numerosity did not significantly correlate with the size of each single visual cue across all trials. This was confirmed using regression analyses that showed that for each participant no relation between a visual cue and numerosity was present (R^2^<0.01 and p>0.08).

The visual properties that were manipulated are: (1) the convex hull (smallest contour around the dot array), (2) the aggregate surface of the dots (or the average diameter of the dots) and (3) density (aggregate surface/convex hull). It was not possible to disentangle average diameter and aggregate surface, when the average diameter increased, aggregate surface also increased. Consequently, the results described below are identical for both visual cues. From here onwards we will therefore only refer to aggregate surface but the same effects hold for average diameter. A priori analyses showed that the different visual cues were strongly correlated. As they were not informative about numerosity across trials, this correlation between different visual cues is not problematic for the task at hand.

### Procedure

First a green fixation cross was shown for 500 ms. Next the first dot-array was presented for 300 ms followed by a blank screen for 1000 ms and a question mark which remained on the screen until the participant responded. Participants had to estimate the number of dots by typing their answer on the numerical keyboard. After the response a blank screen appeared for 1250 to 1500 ms. The stimuli were fully randomized.

### Analyses

**Figure 2:**
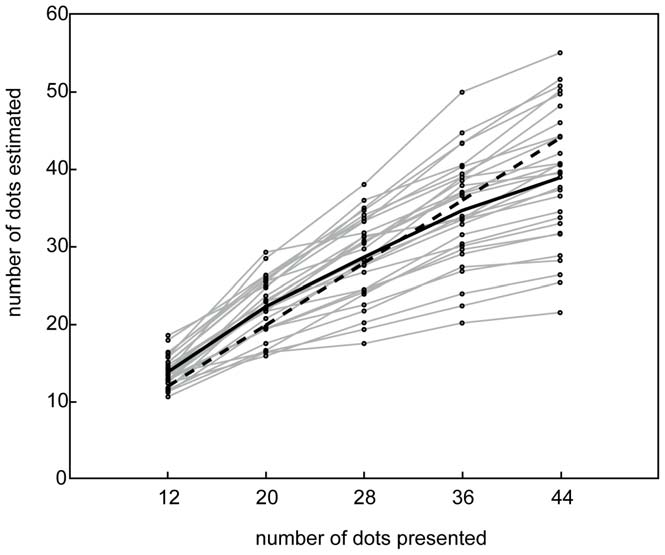
Individual (grey) and average (black solid line) numerical estimates. Approximately half of the participants' consistently over - and half of the participants underestimated the number of dots presented. The dashed line corresponds to the actual values presented.

Outliers (for each participants' responses 2SD larger or smaller than the average estimate) were removed from the data and the average response for each numerosity was calculated (see Figure 2). For reasons of clarity, we will explain our analysis for convex hull but the same analysis was also conducted for density and aggregate surface. First, we divided the stimuli of each target numerosity (12, 20, 28, 36, 44) in two categories: stimuli with a convex hull smaller or larger than the average convex hull. Second, we calculated the participants mean estimate for each category (i.e. small vs. large convex hull). Third, a repeated measures analysis including target numerosity (12, 20, 28, 36, 44) and visual cue size (small or large convex hull) as within participant variables and mean estimate as the dependent variable was conducted. A main effect for visual cue size would indicate that participants' estimates are influenced by the size of the convex hull.

## Results

Similar as in previous studies, a large variation in the individual estimates was present. About half of the subjects overestimated numerosity while the other half underestimated numerosity (see Figure 2).

**Figure 3:**
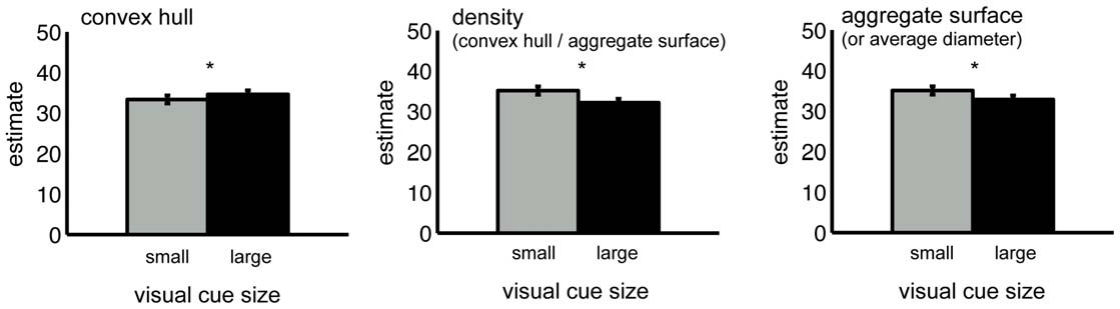
Numerical estimates divided in small and large visual cue sizes. Results show that participants gave larger estimates when dot-arrays had a large convex hull, a low density or a small aggregated surface.

For *convex hull*, the repeated measures analysis showed a significant main effect for target numerosity [*F*(4,112) = 273.91, *p*<0.001]: subjects gave a larger mean estimate for large compared to small numerosities (see Figure 2). Also a significant main effect for visual cue size was present [*F*(1,28) = 9.39, *p* = 0.005] indicating that participants gave a larger estimate for the arrays that were characterized by a relatively large convex hull (see Figure 3). The interaction between target numerosity and visual cue size approached significance [*F*(4,112) = 2.39, *p* = 0.055] implicating that the estimation bias induced by convex hull differed in size between target numerosities. The bias did not systematically increase or decrease with numerosity (the difference in mean estimate was 0.42, −0.08, 1.08, 1.34 and 1.21 dots for respectively target numerosity 12, 20, 28, 36, 44). Post hoc paired samples T-tests showed that this bias was significant for numerosity 12 [*t*(1,28) = −16,89, *p*<0.001], numerosity 28 [*t*(1,28) = −2.77, *p* = 0.01], numerosity 36 [*t*(1,28) = −2.71, *p* = 0.01] but marginally significant for numerosity 44 [*t*(1,28) = −1,99, *p* = 0.056] and not significant for numerosity 20 [*t*(1,28) = 0.2, *p* = 0.84].

For *density*, the results showed a significant main effect for target numerosity [*F*(4,112) = 279.3, *p*<0.001] and for visual cue size [*F*(1,28) = 44.6, *p*<0.001]. These results suggest that the participants gave a larger mean estimate for larger numerosities (see Figure 2) and that this estimate was dependent on density: participants estimated that the number of dots was larger in the arrays that were relatively less dense (see Figure 3). The interaction between target numerosity and visual cue size also reached significance [*F*(4,112) = 4.56, *p* = 0.002]. Again, the estimation bias induced by density differed in size between target numerosities. The bias did not increase or decrease with increasing numerosity (the difference in estimate was 0.94, 2.03, 2.44, 1.24 and 1.55 dots for respectively target numerosity 12, 20, 28, 36 and 44). Post hoc paired samples T-tests showed that the estimation bias induced by density was significant for each target numerosity (all p's<0.019).

For *aggregate surface and average diameter*, the main effect for target numerosity was significant [*F*(4,112) = 273.36, *p*<0.001]. Participants gave a larger mean estimate when the number of dots was larger (see Figure 2). The main effect for visual cue size also reached significance [*F*(1,28) = 41.35, *p*<0.001] indicating that participants estimated the number of dots as larger when the dot array consisted of a relatively small aggregate surface (see Figure 3). Also a significant interaction between target numerosity and visual cue size was obtained [*F*(4,112) = 4.77, *p* = 0.001] suggesting that estimates were biased by aggregate surface but to a different extend for each numerosity (the difference in numerosity estimate was −1.02, −2.06, −2.8, −1.16 and −1.29 dots for respectively target numerosity 12, 20, 28, 36 and 44). Post hoc paired samples T-tests confirmed that the estimation bias induced by aggregate surface (or the average diameter of the dots) was significant for each target numerosity (all p's<0.03).

## Discussion

In this study we investigated the role of visual cues in numerosity estimation. Participants were presented with dot arrays representing 12, 20, 28, 36 or 44 dots and had to estimate the number of dots shown. To investigate the effects of the visual properties of the stimuli on numerosity estimation, we divided the stimuli for each target numerosity into two categories: stimuli with a relatively small convex hull (or density or aggregate surface) and stimuli with a relatively large convex hull (or density or aggregate surface). The results showed that participants' estimates were influenced by the size of the visual cues comprising the dot arrays: participants estimated that the number of dots was larger in the arrays that were characterized by a relatively large convex hull, small density and small aggregate surface (or average diameter). The direction of the bias was comparable to those obtained in a recent numerosity comparison study [8].

The influence of visual cue size on numerosity estimation is remarkable given that the different visual cues separately were not informative about numerosity. No single visual cue increased or decreased consistently with numerosity. The fact that the numerosity estimates were nevertheless influenced by the size of the visual cues suggests that the brain is not equipped with a mechanism that enables humans to estimate numerosity *independently* of its visual cues. It can also be concluded that participants did not rely on a single visual cue but on multiple visual cues when estimating numerosity. Reliance on a single visual cue would not have resulted in numerosity estimates that increased with increasing numerosity [3]. The current results and our previous findings on numerosity comparison suggest that humans integrate multiple visual cues to estimate numerosity [8]. The very poor estimation abilities of humans might therefore not be the result of a poor mapping of approximate number to symbolic number, but of a poor mapping between the mechanism that supports the visual analyses of non-symbolic number images and the symbolic number system. Such an explanation can also account for the finding that participants' estimates improve when they receive feedback [3]. Feedback allows participants to improve their mapping of visual features of the stimuli to symbolic number.

The hypothesis that humans rely on multiple visual cues to judge numerosity has major implications for how researchers currently control the visual cues in numerosity research. The methods to control for the visual cues are grounded in the idea that participants can only rely on a single visual cue throughout the experiment and do not integrate or switch between cues. Instead of designing other, more complicated paradigms (if possible), researchers should question whether controlling visual cues in numerosity studies makes sense. The manipulations of the visual cues are insufficient to control the visual cues and therefore only add noise to the data. More specifically, if participants integrate multiple visual cues to judge number, manipulating the visual cues will not prevent the participants from relying on the visual cues to judge numerosity but instead will increase task difficulty. This is clearly demonstrated in studies that show a decrease in performance when researchers manipulate the visual cues present in the stimuli: human adults can differentiate numerosities that differ with a ratio of 6∶7 when visual cues are controlled for [18] but with a ratio of 7∶8 [19] when visual cues are not controlled for. In contradistinction to our experiments, in daily life the strong relation between numerosity and the majority of visual cues is unlikely to be violated. Consequently, it appears unnecessary to have a brain mechanism that can extract numerosity independently of the visual properties of the stimuli. Researchers might therefore question whether they should use more ecologically valid stimuli that do not control for visual cues to get a true notion of our numerosity estimation or comparison abilities.

Taken together, we show that we rely on visual cues when estimating numerosity. Participants gave a larger estimate for a set of items when it consists of smaller items, a smaller aggregate surface, a larger convex hull or a lower density. This happened even though the visual cues were uninformative; no relation existed between the size of the visual cues and numerosity. These results therefore allowed us to exclude the existence of a mechanism that processes numerosity independently of its visual cues. Consistent with earlier studies on numerosity processing we suggest that humans integrate multiple visual properties present in the stimulus before a number label is attached.
